# Influence of steep Trendelenburg position on postoperative complications: a systematic review and meta-analysis

**DOI:** 10.1007/s11701-021-01361-x

**Published:** 2021-12-31

**Authors:** Satoshi Katayama, Keiichiro Mori, Benjamin Pradere, Takafumi Yanagisawa, Hadi Mostafaei, Fahad Quhal, Reza Sari Motlagh, Ekaterina Laukhtina, Nico C. Grossmann, Pawel Rajwa, Abdulmajeed Aydh, Frederik König, Pierre I. Karakiewicz, Motoo Araki, Yasutomo Nasu, Shahrokh F. Shariat

**Affiliations:** 1 Department of Urology, Comprehensive Cancer Center, Medical University of Vienna, Währinger Gürtel 18-20, 1090 Vienna, Austria; 2 Department of Urology, Okayama University Graduate School of Medicine, Dentistry and Pharmaceutical Sciences, Okayama, Japan; 3 Department of Urology, The Jikei University School of Medicine, Tokyo, Japan; 4 Research Center for Evidence Based Medicine, Tabriz University of Medical Sciences, Tabriz, Iran; 5 Department of Urology, King Fahad Specialist Hospital, Dammam, Saudi Arabia; 6 Men’s Health and Reproductive Health Research Center, Shahid Beheshti University of Medical Sciences, Tehran, Iran; 7 Institute for Urology and Reproductive Health, Sechenov University, Moscow, Russia; 8 Department of Urology, University Hospital Zurich, Zurich, Switzerland; 9 Department of Urology, Medical University of Silesia, 41-800 Zabrze, Poland; 10 Department of Urology, King Faisal Medical City, Abha, Saudi Arabia; 11 Department of Urology, University Medical Center Hamburg-Eppendorf, Hamburg, Germany; 12 Cancer Prognostics and Health Outcomes Unit, Division of Urology, University of Montreal Health Center, Montreal, Canada; 13 Department of Urology, Weill Cornell Medical College, New York, NY USA; 14 Department of Urology, University of Texas Southwestern, Dallas, TX USA; 15 Department of Urology, Second Faculty of Medicine, Charles University, Prague, Czech Republic; 16 Karl Landsteiner Institute of Urology and Andrology, Vienna, Austria; 17 Division of Urology, Department of Special Surgery, Jordan University Hospital, The University of Jordan, Amman, Jordan

**Keywords:** Trendelenburg position, Complication, Thrombosis, Cardiac, Meta-analysis

## Abstract

Intraoperative physiologic changes related to the steep Trendelenburg position have been investigated with the widespread adoption of robot-assisted pelvic surgery (RAPS). However, the impact of the steep Trendelenburg position on postoperative complications remains unclear. We conducted a meta-analysis to compare RAPS to laparoscopic/open pelvic surgery with regards to the rates of venous thromboembolism (VTE), cardiac, and cerebrovascular complications. Meta-regression was performed to evaluate the influence of confounding risk factors. Ten randomized controlled trials (RCTs) and 47 non-randomized controlled studies (NRSs), with a total of 380,125 patients, were included. Although RAPS was associated with a decreased risk of VTE and cardiac complications compared to laparoscopic/open pelvic surgery in NRSs [risk ratio (RR), 0.59; 95% CI 0.51–0.72, *p* < 0.001 and RR 0.93; 95% CI 0.58–1.50, *p* = 0.78, respectively], these differences were not confirmed in RCTs (RR 0.92; 95% CI 0.52–1.62, *p* = 0.77 and RR 0.93; 95% CI 0.58–1.50, *p* = 0.78, respectively). In subgroup analyses of laparoscopic surgery, there was no significant difference in the risk of VTE and cardiac complications in both RCTs and NRSs. In the meta-regression, none of the risk factors were found to be associated with heterogeneity. Furthermore, no significant difference was observed in cerebrovascular complications between RAPS and laparoscopic/open pelvic surgery. Our meta-analysis suggests that the steep Trendelenburg position does not seem to affect postoperative complications and, therefore, can be considered safe with regard to the risk of VTE, cardiac, and cerebrovascular complications. However, proper individualized preventive measures should still be implemented during all surgeries including RAPS to warrant patient safety.

**Supplementary Information:**

The online version contains supplementary material available at 10.1007/s11701-021-01361-x.

## Introduction

During the past few decades, laparoscopic surgery has become the standard procedure in many surgical fields. The quest for improvement for both patients and surgeons using technological innovation has led to widespread use of Intuitive Surgical da Vinci surgical system for pelvic surgery [1]. This technology enables better magnified 3D visualization, tremor filtration, and comfortable remote console [2]. With a marked increase in the use of robotic platforms, a multitude of trials have investigated patients’ benefits regarding oncologic and perioperative outcomes [1]; despite the efforts of researchers, little to no evidence suggests that robot-assisted pelvic surgery (RAPS) improves complication rates and oncologic outcomes over other procedures [3–6]. In addition to these limited benefits, various potential risk and disadvantages to RAPS are likely to result from the steep Trendelenburg position alone or in combination with pneumoperitoneum. This head-down tilt position has been shown to result in decreased lung volume, lung compliance, functional residual capacity and increased peak airway pressure, leading to postoperative pulmonary complications (PPCs). The prevalence of PPCs is approximately 30%, and is associated with increased mortality and morbidity rates [7, 8]. Although its clinical significance remains debatable, this non-physiological positioning typically increase the risk for several intraoperative hemodynamic and intracranial changes, such as increased cardiac contractility, increased cardiac oxygen consumption, and increased intracranial pressure [9, 10]. Compared to research on PPCs, whether these intraoperative changes translate into postoperative detrimental effects remains uncertain. Venous thromboembolism (VTE), which consists of deep vein thrombosis (DVT) and pulmonary embolism, and cardiac and cerebrovascular complications are relatively rare but potentially life-threating. A comprehensive assessment of postoperative complications related to the steep Trendelenburg position is needed to establish preventable measures. Thus, we conducted a systematic review and meta-analysis to clarify the effects of steep Trendelenburg position related to RAPS on postoperative complications.

## Evidence acquisition

This study protocol was registered in the International Prospective Register of Systematic Reviews database (PROSPERO CRD: 42021252198).

### Literature search

This systematic review and meta-analysis was conducted according to the Preferred Reporting Items for Systemic Reviews and Met-Analyses (PRISMA) Protocol 2009 checklist, as shown in Supplementary Table 1 [11]. PubMed, Web of Science, and Cochrane Library databases were searched in January 2021 to identify relevant studies examining the role of RAPS compared to laparoscopic and/or open pelvic surgery for patients with common pelvic malignancies, including prostate, bladder, colorectal, endometrial, and cervical cancers. The following terms were used: (prostate OR bladder OR urothelial OR rectal OR colorectal OR colon OR endometrial OR cervical) AND (cancer OR carcinoma)) AND (robotic surgery OR robot-assisted surgery OR Da Vinci) AND (complication OR morbidity). We also checked the reference lists to detect relevant publications.

### Inclusion and exclusion criteria

The population, intervention, comparator, outcome, and study design (PICOS) approach in this study was as follows: patients with pelvic malignancies (P) who underwent RAPS with curative intent in the steep Trendelenburg position (I) were compared with those who underwent open or laparoscopic pelvic surgery (C) in terms of any grade of VTE, cardiac, and cerebrovascular complications (O) in randomized controlled trials (RCTs) and non-randomized controlled studies (NRSs) (S). Only articles written in English were included in the study. To reduce heterogeneity due to the rarity of objective outcomes, comparative studies that enrolled a minimum of 100 patients in each arm were included. Only studies that performed surgery with a curative intent were included. Studies were excluded if they compared with arms using transanal or transvaginal approach, performed surgery for a benign disease or extraperitoneal RAPS was performed as an intervention group. In case of patient positioning was precisely described, studies comparing extraperitoneal vs transperitoneal RAPS were included. The primary endpoint of interest was VTE, and the secondary endpoints were cardiac and cerebrovascular complications, regardless of the complication grade. Initial screening was performed independently by two investigators based on the titles and abstracts of the articles to identify eligible reports. After the first screening, potentially relevant studies were assessed and reasons for exclusion were noted through a full-text review. Any discrepancies were resolved via consensus with co-authors.

### Data extraction

We extracted the following data from the eligible studies: first author’s name, publication year, period of patient recruitment, recruitment region, study design, surgical procedure, number of patients, age, sex, body mass index (BMI), American Society of Anesthesiology (ASA) score ≥ 3, number of advanced malignancy patients, operative time, number of patients who underwent lymph node (LN) dissection, number of LNs removed, estimated blood loss (EBL), blood transfusion rates, length of stay (LOS), and postoperative complications including VTE, cardiac, and cerebrovascular events. Advanced malignancy was defined as pT ≥ 3 or pathologic stage ≥ 3 (in cases where pT stage is not available). All discrepancies regarding data extraction were resolved via consensus with co-authors.

### Risk of bias assessment

Two investigators independently assessed the risk of bias in each study according to the second edition of the Cochrane Handbook for Systematic Reviews of Interventions. We used the RoB for RCTs and the Risk of Bias In Non-randomized Studies of Interventions (ROBINS-I) for NRSs. (Supplementary Tables 2 and 3, respectively).

### Statistical analyses

A meta-analysis was conducted separately for each dichotomous outcome using the risk ratio (RR) and 95% confidence intervals (CIs). The RCTs were separately analyzed from the NRSs. Continuous variables reported as median and interquartile range were altered to mean and standard deviation (SD) [12]. A random-effects model was applied to represent forest plots in meta-analysis of both RCTs and NRSs and 0.5 continuity corrections for 0 cells were applied. Significant heterogeneity was indicated by a *p* value of < 0.05 in the Cochran’s Q test and a ratio of > 50% in the *I*^2^ statistic. Additionally, considering that the development of VTE is multifactorial with clinically considerable heterogeneity, we performed a meta-regression analysis to explore the potential causes of heterogeneity and estimate the effects of age, BMI, comorbidity (ASA score ≥ 3), advanced malignancy, patients who underwent LN dissection, LN yields, operative time, EBL, and LOS on VTE event rates. Comorbidity, advanced malignancy, and patients who underwent LN dissection were transformed to categorical variables using a cut-off according to the respective median value of 20.2%, 34.0% and 72.7%, respectively. In addition, we performed subgroup analyses of patients according to type of surgical procedures (laparoscopic or open pelvic surgery) in VTE and cardiac complications to reduce and evaluate the effects of pneumoperitoneum and other risk factors. Publication bias was evaluated using Egger’s test, funnel plots were applied for analyses involving more than ten studies. All statistical analyses were performed using Stata^®^/MP 14.0 (Stata Corp., College Station, TX, USA); statistical significance was set at *p* < 0.05.

## Results

Our initial search identified 2991 publications; and 4 additional studies were added after the latest search. After removing duplicate publications, 409 articles were selected for further assessment. After a full-text review, 57 articles with 380,125 patients were deemed eligible for inclusion and exclusion criteria [4–6, 13–66]. A detailed study selection process is shown in Supplementary Fig. 1. The main characteristics of the included studies are summarized in Table 1 and Supplementary Table 4. A total of 10 RCTs and 47 NRSs were identified, with 31 from North America, 12 from Asia, 10 from Europe, 2 from other region, and 2 from international collaborations. Of them, 115,572 (30%), 51,978 (14%), and 212,575 (56%) patients underwent robot-assisted, laparoscopic, and open procedures, respectively. A total of 16 (28%) studies including patients with prostate cancer, 11 (19%) bladder cancer, 11 (19%) colorectal cancer, 10 (18%) endometrial cancer, 4 (7%) uterine cancer, 3 (5%) cervical cancer, and 2 (4%) gynecologic cancers.

**Table 1:** Baseline characteristics of included studies

	Year	Recruitment	Country	Type of surgery	Study design	Total number		Age		Sex (male)		BMI		ASA sore ≥ 3, *n* (%)		pT3 ≥ , *n* (%)	
						Robot	Lapa/open	Robot	lapa/open	Robot	Lapa/open	Robot	Lapa/open	Robot	Lapa/open	Robot	lapa/open
Nix et al. [13]	2010	2008–2009	USA	Bladder cancer	RCT	21	–/20	67.4 (12.7)	–/69.2 (7.8)	14 (67)	–/17 (85)	27.5	–/28.4	NR		3 (14)	–/5 (25)
Asimakopopulos et al. [14]	2011	2007–2008	International	Prostate cancer	RCT	64	64/–	59.6 (5.4)	61.1 (5.1)/–	All		25.8 (2.6)	26.3 (2.2)/–	NR		18 (35)	16 (27)/–
Parekh et al. [15]	2013	2009–2011	USA	Bladder cancer	RCT	20	–/20	68.6 (9.3)	–/65.5 (10.0)	18 (90)	–/16 (80)	27.2 (4.2)	–/28.9 (4.6)	NR		10 (50)	–/7 (35)
Bochner et al. [16]l	2015	2010–2013	USA	Bladder cancer	RCT	60	–/58	65.7 (8.1)	–/64 (8.4)	51 (85)	–/42 (72)	27.9 (1.4)	–/29.0 (2.3)	43 (72)	–/46 (79)	17 (28.3)	–/19 (32.9)
Khan et al. [17]	2016	2009–2012	UK	Bladder cancer	RCT	20	19/20	68.6 (6.8)	68.6 (9.9)/66.6 (8.8)	17 (85)	15 (79)/18 (90)	27.5 (4.2)	26.2 (3.6)/27.4 (3.9)	1 (5)	3 (16)/1 (5)	6(30)	8 (42)/ 6 (30)
Jayne et al. [4]	2017	2011–2014	International	Rectal cancer	RCT	237	234/–	64.4 (11.0)	65.5 (11.9)/–	161 (67.9)	159 (67.9)/–	NR		46 (19)	53 (23)/–	122(51)	122(52)/–
Debakey et al. [18]	2018	2015–2017	Egypt	Rectal cancer	RCT	21	24/–	53.4	50.3/–	11 (52)	13 (54)/–	NR		NR		NR	
Parekh et al. [6]	2018	2011–2014	USA	Bladder cancer	RCT	150	–/152	70	–/67	126 (84)	–/128 (84)	27.9 (4.3)	–/28.3 (5.0)	NR		46 (31)	–/49 (32)
Porpiglia et al. [19]	2018	2010–2011	Italy	Prostate cancer	RCT	60	60/–	63.9 (6.7)	64.7 (5.9)/–	All		26.2 (2.5)	26.8 (2.9)/–	NR		22(37)	22 (37)/–
Silva et al [20]	2018	2015–2017	Brazil	Endometrial cancer	RCT	42	43/–	60 (5.1)	60 (4.8)/–	None		34.8 (7.5)	32.0 (4.1)/–	NR		NR	
Tewari et al. [21]	2003	1999–2002	USA	Prostate cancer	P	200	–/100	59.9	–/63.1	All		27.7	–/27.6	NR		13(7)	–/7 (7)
Boggess et al. [22]	2009	2005–2007	USA	Endometrial cancer	R	103	–/138	61.9 (10.6)	–/64 (12.8)	All		32.9 (7.6)	–/34.7 (9.2)	NR		10	–/20
Krambeck et al. [23]	2009	2002–2005	USA	Prostate cancer	R	294	–/588	59 (6.6)	–/60 (5.9)	All		NR		NR		29 (10)	–/59 (10)
Carlsson et al [24]	2010	2002–2007	Sweden	Prostate cancer	P	1253	–/485	59.3 (6.5)	–/62.5 (5.0)	All		NR		NR		NR	
Doumerc et al.[25]	2010	2006–2008	Australia	Prostate cancer	R	212	–/502	61.3 (9.3)	–/60.1 (6.3)	All		NR		NR		66 (31)	–/177 (35)
Lim et al. [26]	2010	1998–2006 2008–2010	USA	Endometrial cancer	R	122	122/–	62.1 (8.4)	61.6 (11.8)/–	None		31 (8.8)	29.9 (7)/–	NR		NR	
Leitao et al. [27]	2012	2007–2010	USA	Uterine cancer	R	347	302/–	58.3 (10.1)	59.3 (11.0)/–	None		35.5 (8.1)	32.6 (7.0)/–	NR		NR	
Tang et al. [28]	2012	2007–2010	USA	Endometrial cancer	R	129	–/110	59.8 (10.6)	–/58.5 (9.9)	None		39.8 (7.4)	–/40.3 (8.6)	NR		8 (7)	–/9 (8)
Yu et al. [29]	2012	2009	USA	Bladder cancer	R	1144	–/7168	69 (1.5)	–/69 (1.5)	1031 (90.2)	–/6055 (85)	NR		NR		NR	
Froehner et al. [30]	2013	2006–2012	Germany	Prostate cancer	P	317	–/2437	62.6	–/64.9	All		NR		24 (8)	–/281 (12)	76 (24)	–/841(34)
Cardenas-Goicoechea et al. [31]	2013	2003–2010	USA	Endometrial cancer	R	187	245/–	62 (9.4)	61 (10.5)/–	None		31.8 (8.0)	31.8 (8.9)/–	NR		25 (13.3)	31 (12.6)/–
Helvind et al. [32]	2013	2010–2012	USA	Colon cancer	R	101	162/–	72.2 (10.8)	75.3 (9.2)/–	69 (43)	45 (43)/–	25.5 (3.8)	27.7 (5.7)/–	17 (17)	34 (21)/–	NR	
Pilecki et al. [33]	2014	2011	USA	Prostate cancer	R	4374	–/1097	61.7 (7.2)	–/63.1 (7.4)	All		28.6 (4.8)	–/28.5 (5.2)	NR		NR	
Ploussard et al. [34]	2014	2001–2011	France	Prostate cancer	R	1009	1377/–	62.7	62.7/–	All		26.5	26.6/–	NR		424 (42)	562 (40.8)/–
Sugihara et al [35]	2014	2012–2013	Japan	Prostate cancer	R	2126	2483/7202	66.7 (6.7)	67.7 (5.2)/68 (5.9)	All		23.8 (2.6)	23.8 (2.7)/23.8 (2.7)	NR		NR	
Gandaglia et al. [36]	2014	2008–2009	Canada	Prostate cancer	R	353	–/353	69.2 (3.0)	–/69.3 (3.0)	All		NR		NR		152 (43)	–/149 (42)
Moghadamyeghaneh et al. [37]	2015	2009–2012	USA	Rectal cancer	R	872	4737/12,750	64 (12)	62 (13)/64 (13)	556 (65)	2844 (60)/7676 (60)	NR		NR		NR	
Papachristos et al. [38]	2015	2007–2011	Australia	Prostate cancer	R	100	100/–	60.3 (6.0)	60.5 (5.4)/–	All		NR		NR		31 (31)	36 (36)/–
Park et al. [39]	2015	2001–2013	USA	Endometrial cancer	R	350	–/586	58 (10.4)	59.3 (10.6)	None		36.6 (8.4)	36.3 (8.6)/–	NR		22 (6)	–/117 (20)
Wallerstedt et al. [5]	2015	2008–2011	Sweden	Prostate cancer	P	1847	–/778	62.3 (5.9)	–/63 (5.9)	All		26 (2.9)	–/26.3 (2.7)	43 (2)	–/15 (2)	NR	
Zakhari et al. [40]	2015	2008–2012	Canada	Uterine cancer	R	6313	4034/–	NR		None		NR		NR		NR	
Guy et al. [41]	2016	2008–2010	USA	Endometrial cancer	R	1228	–/5914	NR		None		NR		NR		NR	
Ulm et al. [42]	2016	2007–2011	USA	Endometrial cancer	R	165	–/160	64.8 (11.6)	–/64.3 (11.8)	None		34.1 (9.8)	–/35.5 (8.5)	NR		0	–/0
Borgfeldt et al. [43]	2016	2008–2014	Sweden	Uterine cancer	P	430	272/2741	67.2 (11.0)	68.2 (10.5)/68.9 (10.3)	None		29 (7.1)	28.8 (6.5)/28.7 (7.7)	30 (7)	30 (11)/315 (12)	0	0/0
Law et al. [44]	2017	2008–2015	Hong Kong	Rectal cancer	P	220	171/–	63.5 (10.1)	63.3 (13.6)/–	148 (67)	97 (57)/–	median 24.9	24.6/–	47 (21)	35 (20)/–	80 (36)	71 (42)/–
Horovitz et al. [45]	2017	2003–2014	USA	Prostate cancer(Extra- vs transperitoneal surgery)	R	280	340/–	62.3 (6.6)	61.0 (6.8)/–	All		29.0 (4.9)	29.7 (4.7)/–	65 (23)	57 (17)/–	109 (39)	85 (25)/–
Shah et al. [46]	2017	2001–2012	USA	Cervical cancer	R	109	–/202	49.9 (11.7)	–/49.5(12.6)	None		31.3 (6.7)	–/33.1 (6.8)	NR		NR	
Chen et al. [47]	2017	2008–2012	Taiwan	Rectal cancer	R	4744	5578/102858	NR		NR		NR		NR		NR	
Garfinkle et al. [48]	2018	2016	Canada	Rectal cancer	R	154	213/211	61.9 (14)	63.8 (13.3)/63.4 (12.2)	106 (69)	127 (60)/127 (60)	28 (6.1)	27.3 (5.8)/28.7 (6.4)	96 (62)	117 (55)/142 (68)	57 (37)	99 (46)/92 (44)
Nazzani et al. [49]	2018	2008–2013	Canada	Bladder cancer	R	1259	–/8768	NR		972 (77)	–/6804 (78)	NR		NR		NR	
Chen et al. [50]	2019	2014–2018	China	Cervical cancer	R	216	342/–	48.9 (9.7)	47.5 (9.8)/–	None		24.2 (13.4)	23.7 (3.0)/–	NR		NR	
Faraj et al. [51]	2019	2012–2016	USA	Bladder cancer	R	640	–/4921	68.2 (9.3)	–/68.8 (9.7)	NR		28.4 (4.9)	–/28.6 (5.7)	NR		NR	
Piedimonte et al. [52]	2019	2008–2015	USA	Cervical cancer	R	749	–/2584	NR		None		NR		NR		NR	
Flamiatos et al. [53]	2019	2009–2015	USA	Bladder cancer	R	100	–/149	NR		All		27.8 (5.2)	–/28.2 (5,.7)	NR		48 (48)	–/76 (51)
Mukherjee et al. [54]	2019	2010–2015	USA	Prostate cancer	R	52,151	–/16,858	NR		All		NR		NR		NR	
Tang et al. [55]	2019	2010–2016	China	Rectal cancer	R	556	1029/–	57 (11.9)	58 (11.8)/–	347 (62.4)	708 (68.8)/–	23.3 (3.1)	23 (3.1)/–	14 (2.5)	42 (4.1)/–	376 (68)	807 (78)/–
Chen et al. [56]	2019	2012–2016	USA	Bladder cancer	P	143	–/345	70.3 (9.6)	–/69.7 (9.6)	125	–/273	27	–/27.1	113(79)	–/274(79)	50(35)	–/98(28)
Aiko et al. [57]	2020	2013–2018	Japan	Endometrial cancer	R	121	102/–	56 (11)	58 (11)/–	None		25.2 (5.9)	24 (3.8)/–	NR		13 (11)	4 (4)/–
Arora et al. [58]	2020	2007–2019	France	Bladder cancer	R	188	112/–	68.3 (8.1)	67 (9.6)/–	168 (90)	92 (82)	27.0 (4.0)	26.3 (4.9)/–	37 (20)	5 (4)/–	73 (39)	48 (43)/–
Casarin et al. [59]	2020	2008–2015	USA	Uterine cancer/Hysterectomy	R	2536	–/2536	NR		None		NR		NR		NR	
Lo et al. [60]	2020	2012–2016	USA	Colon cancer	R	26,096	28,058/27649	NR		13,204 (51)	9624 (34)/13825(50)	29.1 (0.2)	28.8 (0.1)/28.9 (0.1)	15,535 (60)	16,678 (59)/16443 (59)	NR	
Ye et al. [61]	2020	2015–2019	China	Colorectal cancer/proctectomy	R	293	293/–	60 (12.6)	60 (11.6)/–	168 (57)	170 (58)/–	23.2 (2.5)	23.2 (2.5)/–	45 (16)	36 (12)/–	229 (78)	233 (80)/–
Bedrikovetski et al. [62]	2020	2012–2018	Australia	Rectal cancer	R	177	1269/1980	61 (10.4)	61.8 (12.0)/65 (10.8)	74 (63.2)	735 (57.9)/1313 (66.3)	NR		27 (23)	315 (25)/651 (33)	47 (40)	599 (47)/1002 (52)
Gracia et al. [63]	2020	2012–2016	Spain	Endometrial cancer	P	133	101/–	65.7 (11.9)	63.7 (12.6)	None		28.3 (5.9)	26 (4.4)/–	41 (31)	19 (19)/–	19 (14)	10 (10)/–
Netter et al. [64]	2020	2016–2018	France	Gynecologic cancer	P	175	187/–	Median 61	Median 57.0/–	None		Median 25	Median 24.1/–	NR		NR	
Wang et al. [65]	2020	2014–2017	China	Gynecologic cancer	R	153	123/–	49.7 (9.4)	49.9 (11.1)/–	None		NR		NR		0	0/–
Huang et al. [66]	2021	2017–2018	USA	Prostate cancer	P	376	–/124	62 (8.1)	–/62.7 (6.7)	All		27.7 (3.7)	–/27 (4.4)	NR		158 (43)	–/44 (35)

*RCT* Randomized controlled trial, *P* Prospective study, *R* Retrospective study

There was no publication bias for NRSs in VTE, cardiac, and cerebrovascular complications according to the funnel plot and Egger’s test (*p* = 0.79, *p* = 0.76, and *p* = 0.79, respectively) (Supplementary Fig. 1).

### VTEs and steep Trendelenburg position

**Fig. 1:**
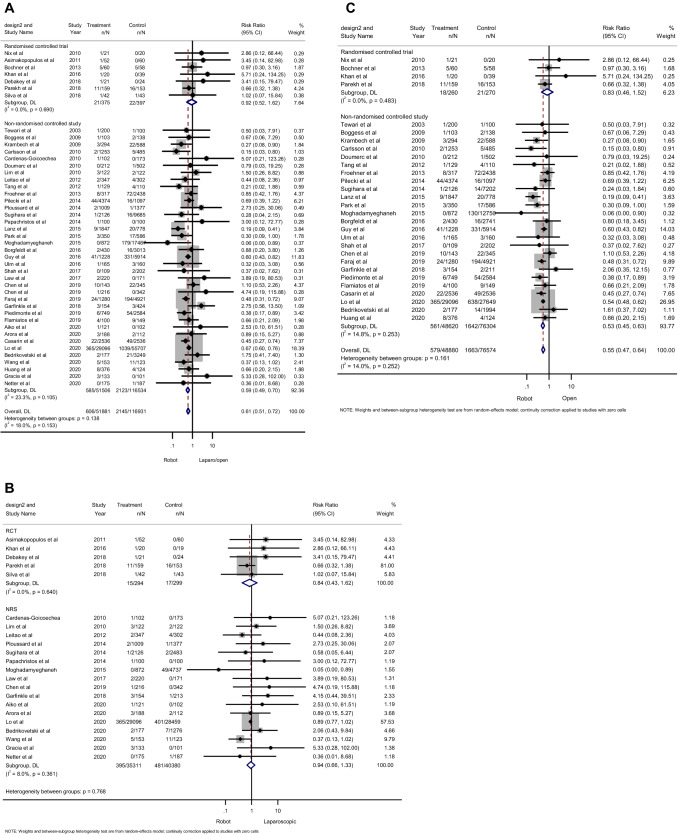
Forest plots for the incidence of venous thromboembolism showing the overall association of robotic surgery (with steep Trendelenburg position) with laparoscopic and open pelvic surgery (**A**), subgroup analyses based on laparoscopic pelvic surgery (**B**), and subgroup analyses based on open pelvic surgery (**C**) in both randomized controlled studies and non-randomized controlled studies

Seven RCTs comprising 772 patients and 37 NRSs with 168,040 patients provided data on the incidence of VTE. Forest plots (Fig. 1A) revealed that there was no significant difference in RCTs (RR 0.92; 95% CI 0.52–1.62; *p* = 0.77), while patients who underwent RAPS had a significantly decreased risk of VTE in NRSs (RR 0.59; 95% CI 0.51–0.72; *p* < 0.001) compared to those who underwent laparoscopic or open surgeries. Based on the Cochran’s *Q* and *I*^2^ tests, no significant heterogeneity was observed in either RCTs (*p* = 0.69, *I*^2^ 0%, respectively) and NRSs (*p* = 0.11, *I*^2^ 23%, respectively). Subgroup analyses based on surgical procedure found that there was no significant difference in laparoscopic pelvic surgery in both RCTs (RR, 0.84; 95%CI, 0.43–1.62; *p* = 0.60) and NRSs (RR 0.94; 95% CI 0.66–1.33; *p* = 0.71) (Fig. 1B); however, there was a statistically significant difference in NRSs (RR 0.53; 95% CI 0.45–0.63; *p* < 0.001), but no significant difference in RCTs (RR 0.83; 95% CI 0.46–1.52; *p* = 0.55) in open pelvic surgery (Fig. 1C).

### Cardiac complications and steep Trendelenburg position

**Fig. 2:**
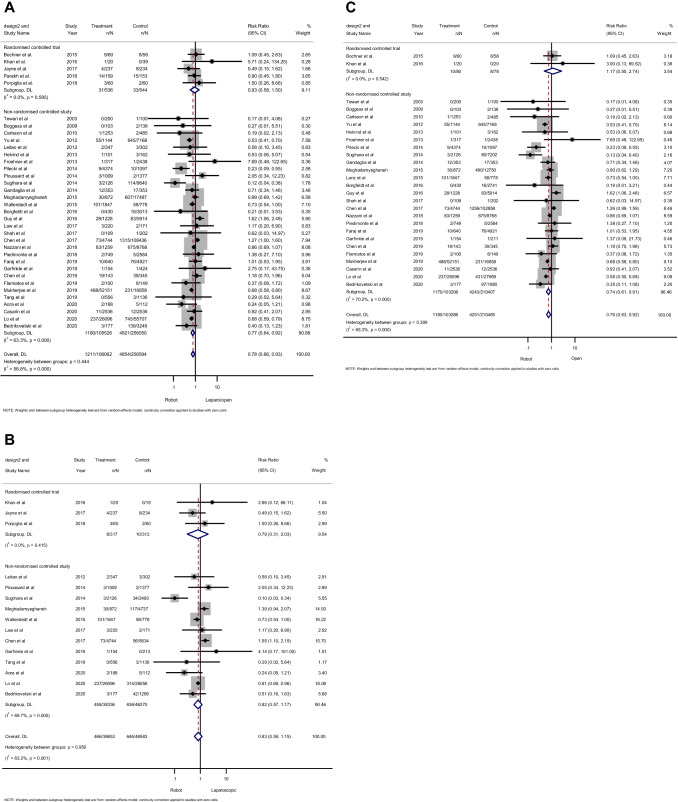
Forest plots for the incidence of cardiac complications showing the overall association of robotic surgery (with steep Trendelenburg position) with laparoscopic and open pelvic surgery (**A**), subgroup analyses based on laparoscopic pelvic surgery (**B**), and subgroup analyses based on open pelvic surgery (**C**) in both randomized controlled studies and non-randomized controlled studies

A total of 5 RCTs comprising 1080 patients and 30 NRSs with 1,361,576 patients provided data on cardiac complications. Forest plots (Fig. 2A) revealed that there was no significant difference in RCTs (RR 0.93; 95% CI 0.58–1.50; *p* = 0.78), while patients who underwent RAPS had a significantly lower risk of cardiac complications (RR 0.77; 95% CI 0.64–0.92; *p* = 0.004) in NRSs compared to those who underwent laparoscopic or open surgeries. Based on the Cochran’s *Q* and *I*^2^ tests, no significant heterogeneity was found in RCTs (*p* = 0.56, *I*^2^ 0%), while there was significant heterogeneity in NRSs (*p* < 0.001, *I*^2^ 63%). Subgroup analysis based on type of surgical procedure (Fig. 2B) revealed no significant difference between RAPS and laparoscopic surgery in both RCTs (RR 0.79, 95% CI 0.31–2.03, *p* = 0.63) and NRSs (RR 0.82, 95%CI 0.57–1.17, *p* = 0.28); meanwhile, there was a statistically significant difference in NRSs (RR 0.74, 95% CI 0.61–0.91, *p* = 0.003), but not in RCTs (RR 1.17, 95% CI 0.50–2.74, *p* = 0.72) in open pelvic surgery (Fig. 2C). Heterogeneities in NRSs were observed in the subgroup analyses of both laparoscopic and open surgery according to the Cochran’s Q test (*p* < 0.001 and *p* < 0.001, respectively) and *I*^2^ test (70% and 70%, respectively).

### Cerebrovascular complications and steep Trendelenburg position

**Fig. 3:**
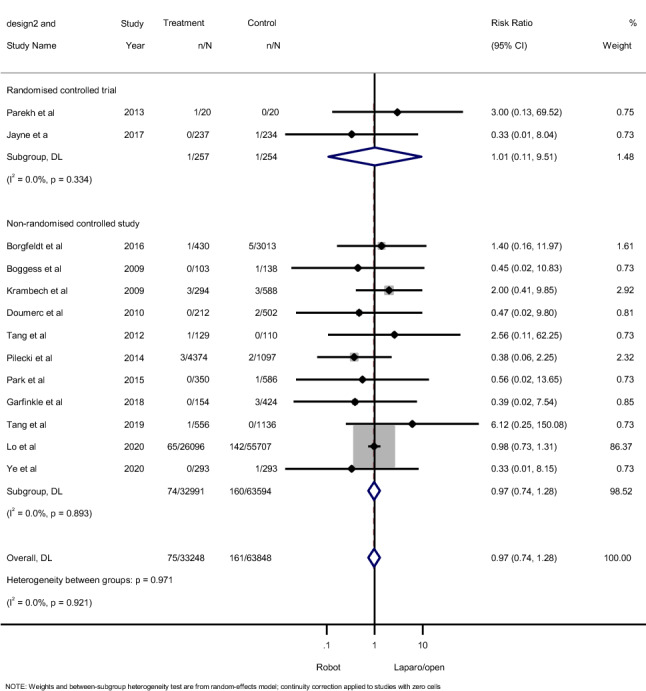
Forest plots for the incidence of cerebrovascular complications showing the overall association of robotic surgery (with steep Trendelenburg position) with laparoscopic and open pelvic surgery in both randomized controlled studies and non-randomized controlled studies

A total of 2 RCTs comprising 511 patients and 11 NRSs with 96,585 patients provided data on cardiac complications. Forest plots (Fig. 3) revealed no significant difference in either RCTs (RR 1.01; 95% CI 0.11–9.51; *p* = 0.99) or NRSs (RR 0.97; 95%CI 0.74–1.28; *p* = 0.83). Based on the Cochran’s *Q* and *I*^2^ tests, there was no significant heterogeneity in RCTs (*p* = 0.33, *I*^2^ 0%), while significant heterogeneity was observed in NRSs (*p* = 0.89, *I*^2^ 0%).

### Heterogeneity exploration

To explore clinically considerable heterogeneity due to the multifactorial etiology of VTE, we performed a meta-regression analysis (Table 2). Among the previously identified potential variables, none of the risk factors exhibited heterogeneity.

**Table 2:** Results of meta-regression analyses for the incidence of VTE

Variable	No. of studies	Regression coefficient	95%CI	*p* value
Cancer type	44	− 0.07303	− 0.1515–0.005459	0.07
Age	35	0.2977	− 0.2790–0.8745	0.30
BMI	25	− 0.3630	− 0.1382–0.6557	0.47
ASA	12	0.7075	− 0.3673–1.7822	0.17
Operative time	28	0.001992	− 0.009763–0.004961	0.18
Estimate blood loss	23	− 0.0000864	− 0.001571–0.001399	0.91
Advanced malignancy	27	0.5684	− 0.002252–1.1391	0.051
LN dissection	22	0.3981	− 0.1590–0.9551	0.15
LN yields	16	0.01573	− 0.02128–0.05274	0.38
LOS	25	0.01064	− 0.007443–0.02872	0.24

*BMI* body mass index, *ASA* the American Society of Anesthesiology, *LN* lymph node, *LOS* length of stay, *CI* confidence interval

## Discussion

In this systematic review and meta-analysis, we investigated the postoperative adverse effects of steep Trendelenburg position of RAPS compared to laparoscopic and open pelvic surgeries. Although the steep Trendelenburg position was associated with a significant risk reduction in the rate of VTE and cardiac complications in NRSs, no difference was found in RCTs between the types of surgical procedures. Additionally, there was no relationship between the steep Trendelenburg position and the risk of cerebrovascular complications.

VTE is a multifactorial disease responsible for significant morbidity and mortality in the postoperative period; patients who experienced VTE after surgery have a 5.3-fold increase in the risk of mortality relative to those who did not [67]. Of the possible mechanisms, venous stasis is one of the key drivers in the development of VTE. The steep Trendelenburg position, described as head tilting of 25–45 degrees downward with leg elevated, facilitates venous return from the lower limbs and decreases blood stasis, which may result in a lower risk of developing intravascular thrombosis [68]. However, there was no significant difference in RCTs despite the presence of statistical significance in NRSs, suggesting that the presence of a steep Trendelenburg position has little to no detrimental effects on postoperative prevalence of VTE; if there is an effect then it is likely to be negligible compared to other risk factors. This is supported by the subgroup analyses that revealed no significant difference between cases with vs those without the steep Trendelenburg position when RAPS was compared to laparoscopic pelvic surgery. There was, however, a significant difference in favor of RAPS compared to open pelvic surgery which is more likely to be associated with VTE risk factors, confounding the real impact of the Trendelenburg position. To date, several risk factors have been shown to be associated with the development of VTE. For example, Trinh et al., who analyzed 2,508,916 patients undergoing eight major surgical oncologic procedures, reported that older age, female sex, severe comorbidity, black race, and insurance status were associated with an increased risk of VTE [67]. Additionally, prolonged operative time, LN dissection, increased BMI, transfusion, and advanced malignancy have been found to be associated with an increased risk of VTE [69, 70]. We assume that these semi-established risk factors affected our analyses, thereby confounding estimates of the VTE risk.

To overcome these inherent heterogeneities caused by confounders, we performed a meta-regression analysis to explore the potential explanations of clinically considerable heterogeneity in VTE driven by various risk factors. The meta-regression, however, confirmed that none of the risk factors were associated with the presence of heterogeneities. Our selection criteria (only comparative studies with ≥ 100 patients per arm were included) may have led to the low heterogeneity in the VTE rate in both RCTs and NRSs.

The steep Trendelenburg position also induces several hemodynamic changes affecting the cardiovascular system. The head-down tilt plus leg raising leads to an increase in central venous pressure ranging from 80 to 305% [71], which increases cardiac preload. Cardiac afterload measured by the systemic vascular resistant index increases at the time of CO_2_ insufflation, followed by a decrease during the steep Trendelenburg position [9]. The intraoperative values of the cardiac output and contractility resulting from these changes remain controversial, varying from no change to a significant increase [72, 73]. Rosendal et al. suggested that they are likely to pose a potential risk for higher cardiac oxygen consumption resulting in adverse cardiac events [71]. Despite these concerns, there was no difference between two types of surgical procedures in RCTs and subgroup analyses based on laparoscopic surgery, suggesting that the steep Trendelenburg position provides little to no impact on postoperative cardiac complications. Although a considerable relative risk reduction was observed in NRSs of cardiac complications as well as VTE (23% and 39% risk reduction, respectively), interpretations should be done with caution due to the influences of other uncontrolled confounding factors.

We also evaluated the association between the steep Trendelenburg position and cerebrovascular complications. This positioning, combined with pneumoperitoneum, has been shown to increase intracranial pressure, thereby reducing cerebral perfusion pressure (CPP) resulting in cerebral ischemia [74]. Our findings, however, showed that the steep Trendelenburg position has only negligible impact on the likelihood of postoperative cerebrovascular complications. One potential reason for this could be a compensation through a concomitant increase in mean arterial pressure for any increase of intracranial pressure, thereby maintaining CPP and cerebral oxygen saturation [10, 75, 76].

Our meta-analysis has several limitations. First, this study includes considerable heterogeneity primarily due to the included different types of surgeries. We attempted to explain the clinically considerable heterogeneity in VTE risk by assessing the association between VTE risk and other risk factors. Although we performed meta-regression and subgroup analyses, we could not explore within-study heterogeneity, which is a limitation inherent to meta-regression analysis. The unaccounted variable use of VTE prophylaxis may affect the prevalence of thromboembolism. However, included studies made little mention of measures for VTE prevention, which could not be analyzed using meta-regression. Additionally, the duration, inclination angle and practical techniques of steep Trendelenburg varied across studies, centers, surgeons. Indeed, Souki et al. described that only 2.1% of assessed institutions had a policy on the safe limits of positioning during the steep Trendelenburg position [77]. In general, the Trendelenburg angle in laparoscopic pelvic surgery may not be steep, but the variable angles of this positioning are required depending on the type of surgery, which could affect the findings of our study. Furthermore, due to the lack of standardized follow-up management, the introduction of early ambulation as well as perioperative treatment strategies including radiotherapy and chemotherapy could not be accounted for.

We found no detrimental effects related to steep Trendelenburg on postoperative complications. In addition, RAPS was associated with a significantly lower risk of VTE and cardiac complications compared to open pelvic surgery. There are concerns regarding the generalizability of these data and a need for well-designed prospective studies with long follow-up. Hypotheses such as the one suggesting that prolonged Trendelenburg position may cause postoperative cognitive decline need to be adequately assessed [78]. The European Association of Urology Robotic Urology Section Scientific Working Group recommends, indeed, prolonged postoperative use (four weeks) of low molecular weight heparin with 100% of agreement for patients performing robot-assisted radical cystectomy [79]. Rigorous methodology, scientifical and critical verification of effectiveness of the robotic platform should be continued.

## Conclusion

We found that the steep Trendelenburg position has negligible impacts on postoperative thromboembolic, cardiac, and cerebrovascular complications. However, appropriate preventive measures against these complications should be implemented. The individual risk for each patient according to his general health, tumor characteristics, and peri- and intraoperative history should guide preventative measures, especially when RAPS is performed.

## Supplementary Information

Below is the link to the electronic supplementary material.

Supplementary file1 Supplementary Figs. 1. Flow diagram of the study selection for the systematic review and meta-analysis. Supplementary Fig. 2. Funnel plots and the results of Egger’s tests of included non-randomized controlled studies performing meta-analyses for venous thromboembolism (A), cardiac complications (B), and cerebrovascular complications (C). (DOCX 52 KB)

Supplementary file2 (DOC 66 KB)

Supplementary file3 (DOCX 54 KB)
