# Supplementary material for: Influence of steep Trendelenburg position on postoperative complications: a systematic review and meta-analysis
Source: J Robot Surg. 2021 Dec 31;16(6):1233–47. doi: 10.1007/s11701-021-01361-x (PMC9606098; doi:10.1007/s11701-021-01361-x)
Supplement: Supplementary file 1 — Supplementary file1 Supplementary Figs. 1. Flow diagram of the study selection for the systematic review and meta-analysis. Supplementary Fig. 2. Funnel plots and the results of Egger’s tests of included non-randomized controlled studies performing meta-analyses for venous thromboembolism (A), cardiac complications (B), and cerebrovascular complications (C). (DOCX 52 KB) [file 11701_2021_1361_MOESM1_ESM.docx]

**Supplementary Figure. 1**

**Records identified through PubMed, Web of Science, Cochrane:**

**Search Query:**

(prostate OR bladder OR urothelial OR rectal OR colorectal OR colon OR endometrial OR cervical) AND (cancer OR carcinoma)) AND (robotic surgery OR robot-assisted surgery OR Da Vinci) AND (complication OR morbidity)

**(n = 2991)**

**Records screened after duplicates removed**

**(n = 2995)**

Records excluded (n = 2586)

Not in the field of interest (n = 1965)

Book (n = 5)

Review article (n = 446)

Case report (n = 114)

Abstract only (n = 19)

Other than English (n = 37)

Additional record identified from further up-to-date search

**(n = 4)**

**Identification**

**Screening**

**Eligibility**

**Included**

**Full-text articles assessed for eligibility**

**(n = 409)**

Articles excluded after evaluation (n = 352)

Does not meet our inclusion criteria

(n = 351)

Overlapping study population (n = 1)

**Articles included in quantitative synthesis**

**(n = 59)**

**Articles included in this meta-analysis**

**(n = 57)**

**Supplementary Figure 2A. VTE**

Egger’s test p=0.79

**Supplementary Figure 2B. Cardio**

Egger’s test p=0.76

**Supplementary Figure 2C. Cerebrovascular**

Egger’s test p=0.79
