# Supplementary material for: Influence of steep Trendelenburg position on postoperative complications: a systematic review and meta-analysis
Source: J Robot Surg. 2021 Dec 31;16(6):1233–47. doi: 10.1007/s11701-021-01361-x (PMC9606098; doi:10.1007/s11701-021-01361-x)
Supplement: Supplementary file 3 — Supplementary file3 (DOCX 54 KB) [file 11701_2021_1361_MOESM3_ESM.docx]

**Supplementary Table 2.**

Risk of bias summary of the included studies for systematic review and meta-analysis

(A) Random sequence generation (selection bias); (B) allocation concealment (selection bias); (C) blinding of participants and personal (performance bias); (D) blinding of outcome assessment (detection bias); (E) incomplete outcome data (attrition bias); (F) selective reporting (reporting bias); (G) other bias. Green circles represent a low risk of bias and confounding, red circles represent a high risk of bias and confounding, and yellow circles represent an unclear risk of bias and confounding.

| **Author, year** | **A** | B | C | D | E | F | G |
| --- | --- | --- | --- | --- | --- | --- | --- |
| **Nix 2010** | 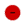 | 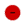 | 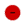 | 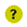 | 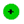 | 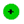 | 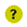 |
| **Ashimakopopulous 2011** | 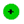 | 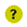 | 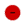 | 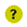 | 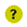 | 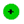 | 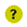 |
| **Parekh 2013** | 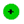 | 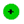 | 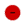 | 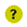 | 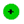 | 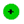 | 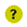 |
| **Bochner 2015** | 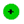 | 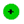 | 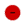 | 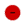 | 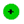 | 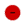 | 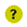 |
| **Khan 2016** | 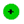 | 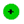 | 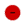 | 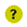 | 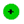 | 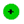 | 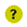 |
| **Jayne 2017** | 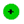 | 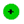 | 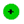 | 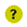 | 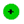 | 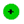 | 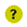 |
| **Debakey 2018** | 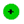 | 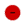 | 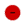 | 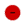 | 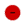 | 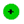 | 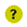 |
| **Parekh 2018** | 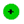 | 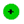 | 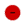 | 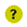 | 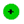 | 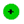 | 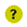 |
| **Porpiglia 2018** | 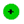 | 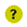 | 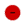 | 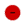 | 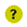 | 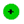 | 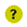 |
| **Silva 2018** | 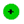 | 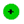 | 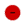 | 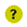 | 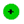 | 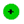 | 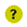 |

**Supplementary Table 3.** Risk of bias assessment for individual studies using the Risk of Bias in non-randomized controlled studies-of interventions tool (ROBINS-I).

| **Author** | **Confounding** | **Participant selection** | **Classification of interventions** | **Deviations from intended intervention** | **Missing data** | **Measurement of outcomes** | **Selection of the reported result** | **Overall** |
| --- | --- | --- | --- | --- | --- | --- | --- | --- |
| Tewari 2003 | Serious | Serious | Low | Low | Low | Serious | Low | Serious |
| Boggess 2009 | Serious | Moderate | Low | Moderate | Low | Low | Low | Serious |
| Krambech 2009 | Serious | Serious | Low | Low | Low | Serious | Low | Serious |
| Carlsson 2010 | Moderate | Low | Low | Low | Low | Low | Low | Moderate |
| Doumerc 2010 | Serious | Serious | Low | Low | Low | Serious | Low | Serious |
| Lim 2010 | Serious | Moderate | Low | Low | Low | Low | Low | Serious |
| Leitao 2012 | Moderate | Moderate | Low | Moderate | Low | Low | Low | Moderate |
| Tang 2012 | Serious | Serious | Low | Low | Moderate | Low | Low | Serious |
| Yu 2012 | Low | Low | Low | Low | Moderate | Moderate | Low | Moderate |
| Froehner 2013 | Serious | Moderate | Low | Low | Moderate | Moderate | Low | Serious |
| Cardenas-Goicoechea 2013 | Serious | Serious | Low | Moderate | Low | Serious | Low | Serious |
| Helvind 2013 | Serious | Serious | Low | Moderate | Serious | Serious | Low | Serious |
| Pilecki 2014 | Serious | Serious | Low | Moderate | Serious | Moderate | Low | Serious |
| Ploussard 2014 | Moderate | Low | Low | Low | Low | Moderate | Low | Moderate |
| Sugihara 2014 | Serious | Moderate | Low | Moderate | Serious | Serious | Low | Serious |
| Gandaglia 2014 | Serious | Serious | Low | Moderate | Serious | Serious | Low | Serious |
| Moghadamyeghaneh 2015 | Serious | Serious | Low | Moderate | Serious | Serious | Low | Serious |
| Papachristos 2014 | Serious | Serious | Low | Low | Low | Serious | Low | Serious |
| Park 2015 | Serious | Serious | Low | Moderate | Moderate | Serious | Low | Serious |
| Wallerstedt　2015 | Low | Low | Low | Serious | Moderate | Moderate | Low | Serious |
| Zakhari 2015 | Serious | Serious | Low | Moderate | Serious | Serious | Moderate | Serious |
| Guy 2016 | Serious | Serious | Low | Moderate | Serious | Serious | Moderate | Serious |
| Ulm 2016 | Serious | Serious | Low | Low | Low | Serious | Moderate | Serious |
| Borgfeldt 2016 | Serious | Serious | Low | Moderate | Moderate | Serious | Low | Serious |
| Law 2017 | Serious | Moderate | Low | Low | Low | Serious | Low | Serious |
| Horovitz 2017 | Low | Serious | Low | Moderate | Low | Moderate | Low | Serious |
| Shah 2017 | Serious | Serious | Low | Moderate | Serious | Serious | Low | Serious |
| Chen 2017 | Serious | Moderate | Low | Moderate | Serious | Moderate | Serious | Serious |
| Garfinkle 2018 | Serious | Serious | Low | Moderate | Serious | Serious | Moderate | Serious |
| Nazzani 2018 | Low | Low | Low | Low | Low | Moderate | Serious | Serious |
| Chen 2019 | Serious | Serious | Low | Low | Low | Serious | Low | Serious |
| Faraj 2019 | Serious | Serious | Low | Moderate | Low | Serious | Serious | Serious |
| Piedimonte 2019 | Serious | Serious | Low | Serious | Moderate | Serious | Serious | Serious |
| Flamiatos 2019 | Serious | Serious | Low | Moderate | Low | Low | Low | Serious |
| Mukherjee 2019 | Serious | Serious | Low | Serious | Low | Serious | Serious | Serious |
| Tang 2019 | Serious | Serious | Low | Moderate | Low | Serious | Low | Serious |
| Chen 2019 | Moderate | Low | Low | Moderate | Low | Moderate | Low | Moderate |
| Aiko 2020 | Serious | Serious | Low | Low | Low | Serious | Low | Serious |
| Arora 2020 | Serious | Serious | Low | Moderate | Low | Serious | Low | Serious |
| Cassarin 2020 | Serious | Serious | Low | Moderate | Moderate | Serious | Low | Serious |
| Lo 2020 | Serious | Serious | Low | Moderate | Moderate | Serious | Low | Serious |
| Ye 2020 | Low | Serious | Low | Low | Low | Serious | Low | Serious |
| Bedrikovetski 2020 | Serious | Serious | Low | Low | Serious | Serious | Low | Serious |
| Gracia 2020 | Serious | Serious | Low | Low | Moderate | Low | Low | Serious |
| Netter 2020 | Low | Moderate | Low | Low | Low | Moderate | Low | Moderate |
| Wang 2020 | Serious | Serious | Low | Low | Moderate | Serious | Low | Serious |
| Huang 2021 | Low | Low | Low | Low | Low | Low | Low | Low |

**Supplementary Table 4. Perioperative outcomes of included studies**

|  | Operative time  mean, (SD) | | LN dissection  n, (%) | | Number of LN removed  n, (SD) | | | EBL  mean, (SD) | | Transfusion  n, (%) | | Length of stay  mean, (SD) | |
| --- | --- | --- | --- | --- | --- | --- | --- | --- | --- | --- | --- | --- | --- |
|  | **Robot** | **Lapa/Open** | **Robot** | **Lapa / Open** | **Robot** | **Lapa** | **Open** | **Robot** | **Lapa/Open** | **Robot** | **Lapa/Open** | **Robot** | **Lapa/Open** |
| Nix et al [13] | **252** | **- / 210** | **All** | | **20 (4.8)** | **-** | **19 (5.9)** | **258** | **- /576** | **NR** | | **5.1** | **- / 4** |
| Asimakopopulos et al [14] | **NR** | | **NR** | | **NR** | | | **NR** | | **0 (0)** | **3 (5) / -** | **NR** | |
| Parekh et al [15] | **302 (101)** | **- / 282 (64.9)** | **All** | | **11 (9.4)** | **- / -** | **23 (9.6)** | **488 (343)** | **- / 775 (537)** | **8 (40)** | **- / 10 (50)** | **6** | **- / 6** |
| Bochner et al  [16] | **456 (82)** | **- / 329 (77)** | **All** | | **19.5 (10) / 31.9 (12)??** | **- / -** | **18.9 (10) /30.0 (12)** | **500 (437)** | **- / 681 (328)** | **NR** | | **8** | **- / 8** |
| Khan et al  [17] | **398 (98)** | **301 (51) / 293 (66)** | **NR** | | **16.3** | **15.5/18.8** | **18.8** | **585 (618)** | **460 (485) / 808 (329)** | **NR** | | **11.9 (6.2)** | **9.7 (3.6) / 14.4 (5.9)** |
| Jayne et al  [4] | **299 (88)** | **261 (83) / -** | **NR** | | **23.2 (12.0)** | **24.1 (12.9)** | **-** | **NR** | | **NR** | | **8** | **8.2 / -** |
| Debakey et al [18] | **NR** | | **NR** | | **NR** | | | **NR** | | **NR** | | **5.5 (3.2)** | **4.3 (2.3)** |
| Parekh et al  [6] | **441.8 (50)** | **363.3 (31.9)** | **149 (94)** | **- / 152 (99)** | **23.3 (12.5)** | **- / 25.7 (14.5)** | **25.7(14.5)** | **333 (222)** | **- / 700 (370)** | **35 (22)** | **- / 65 (42)??** | **6.8 (2.7)** | **7.5 (0.8)** |
| Porpiglia et al [19] | **NR** | | **13 (22)** | **- / 13 (22)** | **17 (7.4)** | **17 (4.4)** | **-** |  |  | **NR** | | **NR** | |
| Silva et al [20] | **333 (80)** | **282 (69)/ -** | **NR** | | **25.5 (13.3)** | **19.5 (6.9)** | **-** | **825 (670)** | **426 (337) / -** | **NR** | | **3.3 (0.7)** | **12.8 (9.4)** |
| Tewari et al [21] | **160 (181)** | **- /163 (231)** | **NR** | | **NR** | | | **153 (133)** | **- / 1755 (960)** | **0 (0)** | **- /67 (67)** | **2.1 (0.7)** | **- / 4 (0.6)** |
| Boggess et al [22] | **191 (36)** | **- / 147(49)** | **NR** | | **32.9 (26.2)** | **-** | **14.9 (11.3)** | **75 (101)** | **- / 266 (185)** | **1 (1)** | **- / 2 (1)** | **1.0 (0.2)** | **- / 4.4 (2.0)** |
| Krambech et al [23] | **242 (60)** | **- / 211(79)** | **All** | | **NR** | | | **NR** | | **15 (5)** | **- / 77 (13)** | **NR** | |
| Carlsson et al [24] | **NR** | | **38 (3)** | **- / 68 (14)** | **NR** | | | **NR** | | **58 (5)** | **- /112 (23)** | **NR** | |
| Doumerc et al [25] | **192 (73)** | **- / 148 (42)** | **158 (75)** | **- / 239 (48)** | **NR** | | | **NR** | | **2 (1)** | **- / 10 (2)** | **3.7 (0.9)** | **6 (1.2)** |
| Lim et al  [26] | **147 (48)** | **187 (60) / -** | **NR** | | **25.1 (12.7)** | **43.1 (17.8)** | **-** | **81 (46)** | **207 (109) / -** | **0 (0)** | **3 (2) / -** | **1.5 (0.9)** | **3.2 (2.3) / -** |
| Leitao et al [27] | **336 (81)** | **281 (60) / -** | **NR** | | **20.5** | **22.5** | **-** | **125 (68)** | **376 (157) / -** | **1 (0)** | **1 (0) / -** | **1.8 (0.9)** | **5 (2.4)** |
| Tang et al [28] | **237 (65)** | **- / 177 (50)** | **97 (75.2)** | **- / 82 (74.5)** | **13.0 (11.3)** | **-** | **10.7 (11.3)** | **160 (150)** | **- / 292 (226)** | **6 (5)** | **- / 8 (7)** | **1.5 (1.0)** | **- / 4.1 (2.2)** |
| Yu et al  [29] | **NR** | | **983 (86.3)** | **- / 4954 (69.1)** | **NR** | | | **NR** | | **351 (30.7)** | **- / 2966 (41)** | **11.4 (5.7)** | **10.9 (4.1) / -** |
| Froehner et al  [30] | **NR** | | **290 (92)** | **- / 2324 (95)** | **13** | **- / 15** | **15** | **NR** | | **24 (8)** | **- /182 (7)** | **8** | **- / 7.7** |
| Cardenas-Goicoechea et al[31] | **218 (59)** | **161 (59) / -** | **All** | | **18.9 (10.4)** | **20 (12.1)** | **-** | **110 (83)** | **187 (169)** | **5 (3)** | **5 (2)** | **1.9 (1.7)** | **2.31 (2.2)** |
| Helvind et al [32] | **243 (62)** | **254 (117) / -** | **NR** | | **27.7 (10.8)** | **27.6 (10.5)** | **-** | **NR** | | **NR** | | **10.0 (5.0)** | **19.6 (11.8) / -** |
| Pilecki et al [33] | **212 (74)** | **- / 174 (85)** | **NR** | | **NR** | | | **NR** | | **82 (2)** | **- / 194 (18)** | **NR** | |
| Ploussard et al [34] | **129** | **176 / -** | **458 (45)** | **- / 603 (44)** | **NR** | | | **515** | **800 / -** | **29 (2.9)** | **65 (4.7) / -** | **4.0** | **5.7** |
| Sugihara et al [35] | **NR** | | **NR** | | **NR** | | | **NR** | | **15 (1)** | **56 (2) / 523 (7)** | **11** | **11** |
| Gandaglia et al [36] | **NR** | | **230 (65.2)** | **- / 283 (80.2)** | **NR** | | | **NR** | | **8 (2.3)** | **- / 29 (8.2)** | **1.0 (0.7)** | **2.3 (0.7)** |
| Moghadamyeghaneh et al [37] | **NR** | | **NR** | | **NR** | | | **NR** | | **NR** | | **8 (6)** | **8 (6) / 10 (7)** |
| Papachristos et al [38] | **215 (38)** | **201 (37) / -** | **8 (8)** | **10 (10) / -** | **NR** | | | **413 (190)** | **388 (170) / -** | **0** | **1 (1) / -** | **2.7 (3.0)** | **3.3 (4.4) / -** |
| Park et al [39] | **227 (63)** | **- / 258 (79)** | **250 (71)** | **- / 483 (82)** | **pelvic 11.0 para-aorta 4.1** | **-** | **pelvic 11.3 para-aorta 4.1** | **428 (256)** | **- / 1463 (806)** | **9 (3)** | **- / 102 (17)** | **2.4 (0.9)** | **- / 27.8 (16.3)** |
| Wallerstedt et al [5] | **175 (39)** | **- / 103 (194)** | **225 (12)** | **- / 203 (26)** | **NR** | | | **185 (111)** | **- / 683 (333)** | **NR** | | **3 (1.5)** | **- / 4 (1.5)** |
| Zakhari et al [40] | **NR** | | **4613 (73.1)** | **2664 (66) / -** | **NR** | | | **NR** | | **512 (8.1)** | **328 (8.1) / -** | **NR** | |
| Guy et al [41] | **NR** | | **973 (79)** | **- /4205 (71.1)** | **NR** | | | **102 (103)** | **- / 237 (221)** | **41 (3)** | **- / 739 (12)** | **2 (2.1)** | **- / 5.1 (4.9)** |
| Ulm et al [42] | **NR** | | **All** | | **para-aorta 1.0 (1.8) pelvic 6.4 (4.2)** | **-** | **para-aorta 1.6 (2.3) pelvic 8.7 (7.4)** | **102 (103)** | **- / 237 (221)** | **NR** | | **1.4 (1.2)** | **- / 3 (1.8)** |
| Borgfeldt et al  [43] | **without LN removed 117 (54) with LN removed 204 (68)** | **without LN removed 120 (46)/ 108 (57) with LN removed 279 (86) / 191 (83)** | **222 (52) / NR** | **11 (4) / 727 (27)** | **NR** | | | **without LN removed 95 (294) with LN removed 87 (205)** | **without LN removed 113 (182) / 266 (454) with LN removed 283 (181) / 377 (379)** | **3 (1)** | **4 (1)/161 (6)** | **without LN removed 1.3 (6.5) with LN removed 2.4 (3.0)** | **without LN removed 2.3 (2.5) /3.9 (6.1) with LN removed 6.3 (4.6) / 5.8 (4.1)** |
| Law et al  [44] | **332 (96)** | **268 (78) / -** | **NR** | | **median 14** | **median 12 / -** | **-** | **678 (450)** | **550 (371)/ -** | **NR** | | **19.5 (11.2)** | **24.3 (15.1)** |
| Horovitz et al [45] | **198 (45)** | **189 (54) / -** | **257 (92)** | **165 (49) / -** | **NR** | | | **191 (125)** | **211 (142) / -** | **NR** | | **1.3 (1.1)** | **1.1 (0.5)** |
| Shah et al [46] | **mean 257 (range129-352)** | **NR** | **All** | | **19 (8.3)** | **-** | **29.3 (13.7)** | **106** | **- / 483** | **0 (0)** | **- / 8 (4)** | **2.9 (1.4)** | **26.9 (17.5)** |
| Chen et al [47] | **NR** | | **NR** | | **NR** | | | **NR** | | **NR** | | **6.5 (3.4)** | **4.8 (2.6) / 6.5 (3.4)** |
| Garfinkle et al [48] | **296 (115)** | **296 (118) / 269 (113)** | **NR** | | **15.6 (7.6)** | **16.6 (7.4)** | **16.9 (8.8)** | **NR** | | **NR** | | **median 6.0** | **median 5.0 / 4.0** |
| Nazzani et al [49] | **NR** | | **1148 (91.2)** | **- / 7207 (82.2)** | **NR** | | | **NR** | | **244 (19)** | **- / 3069 (35)** | **8.3 (3.0)** | **- / 9 (3.7)** |
| Chen et al [50] | **197 (58)** | **234 (60) / -** | **NR** | | **23.5 (9.3)** | **25.1 (11.4)** | **-** | **163 (321)** | **281 (247) / -** | **8 (4)** | **39 (11) / -** | **10.9 (4.4)** | **12.3 (5.1)** |
| Faraj et al [51] | **NR** | | **NR** | | **NR** | | | **NR** | | **6 (1)** | **- / 74 (2)** | **8.0 (6.1)** | **- / 9.7 (7.5)** |
| Piedimonte et al [52] | **NR** | | **125 (17)** | **- / 57 (22)** | **NR** | | | **NR** | | **NR** | | **median 2** | **median 4** |
| Flamiatos et al  [53] | **411 (70)** | **- / 371 (102)** | **NR** | | **NR** | | | **166.7 (111)** | **- / 717 (630)** | **75 (50)** | **- / 11 (11)** | **5.7 (3.0)** | **- / 6.7 (2.2)** |
| Mukherjee et al  [54] | **NR** | | **NR** | | **NR** | | | **NR** | | **720 (1.38)** | **- / 1291 (8)** | **1.9 (0)** | **2.8 (0)** |
| Tang et al [55] | **222 (57)** | **185 (54) / -** | **NR** | | **14.9 (5.3)** | **13.7 (5.9)** | **-** | **67.5 (34.3)** | **74 (32.5) / -** | **20 (36)** | **48 (4.2) / -** | **11.4 (5.7)** | **10.9 (4.1) / -** |
| Chen et al [56] | **436 (76)** | **- /328 (80)** | **NR** | | **42 (16.3)** | **-** | **43 (17.8)** | **217 (98)** | **- / 500 (296)** | **30 (21)** | **- /141(41)** | **5.8 (2.6)** | **- / 4.5 (0.3)** |
| Aiko et al [57] | **178 (41)** | **133 (28) / -** | **119 (98)** | **97 (95) / -** | **41 (16)** | **42 (17)** | **-** | **237 (146)** | **196 (153) / -** | **0** | **0 (0) / -** | **8 (3)** | **9 (4) / -** |
| Arora et al [58] | **262 (59)** | **270 (44) / -** | **175 (93)** | **98 (88) / -** | **NR** | | | **692 (370)** | **567 (296) / -** | **79 (42)** | **34 (30) / -** | **median13** | **14** |
| Casarin et al  [59] | **228 (96)** | **- / 198 (90)** | **322 (13)** | **- / 1896 (75)** | **NR** | | | **NR** | | **139 (6)** | **- / 307 (12)** | **3.2 (3.1)** | **- / 3.3 (1.5)** |
| Lo et al [60] | **209 (2.1)** | **169 (0.6) / 162 (1.2)** | **NR** | | **NR** | | | **NR** | | **NR** | | **4.6 (0.1)** | **5.1 (0.03) / 7.0 (0.7)** |
| Ye et al [61] | **171 (42)** | **145 (42) / -** | **NR** | | **16.0 (3.8)** | **15.7(3.7)** | **-** | **106 (114)** | **138 (111) / -** |  |  | **9.1** | **9.2** |
| Bedrikovetski et al  [62] | **NR** | | **NR** | | **20 (2.7)** | **28.3 (12.6)** | **21.8 (3.4)** | **NR** | | **NR** | | **25.3 (14.7)** | **29.5 (14.9) / 130.5 (73.4)** |
| Gracia et al  [63] | **NR** | | **60(45)** | **49 (49) / -** | **NR** | | | **60, 100, 87.5** | **100, 105, 180** | **4 (3)** | **4 (4)/ -** | **3, 3, 2** | **3, 3, 4** |
| Netter et al  [64] | **median 163 (95%CI 171-194)** | **median 137 (95%CI140-162) / -** | **NR** | | **NR** | | | **NR** | | **NR** | | **median 2 (95%CI 1.8-2.2)** | **1.0 (1.4-1.9) / -** |
| Wang et al [65] | **280 (135)** | **260 (115) / -** | **NR** | | **29 (11)** | **17 (27)** | **-** | **150 (200)** | **200 (200) / -** | **NR** | | **NR** | |
| Huang et al [66] | **183 (49)** | **- /113 (34)** | **312 (83)** | **- / 106 (85)** | **10.3 (7.4)** | **-** | **8.2 (5.2)** | **233 (185)** | **- / 150 (74)** | **2 (1)** | **- / 0 (0)** | **1 (0)** | **- / 1 (0)** |
